# Serum creatinine elevation after renin-angiotensin system blockade and long term cardiorenal risks: cohort study

**DOI:** 10.1136/bmj.j791

**Published:** 2017-03-09

**Authors:** Morten Schmidt, Kathryn E Mansfield, Krishnan Bhaskaran, Dorothea Nitsch, Henrik Toft Sørensen, Liam Smeeth, Laurie A Tomlinson

**Affiliations:** 1 Department of Non-Communicable Disease Epidemiology, London School of Hygiene and Tropical Medicine, London, UK; 2 Department of Clinical Epidemiology, Aarhus University Hospital, Aarhus, Denmark; 3 Department of Internal Medicine, Regional Hospital of Randers, Randers, Denmark

## Abstract

**Objective** To examine long term cardiorenal outcomes associated with increased concentrations of creatinine after the start of angiotensin converting enzyme inhibitor/angiotensin receptor blocker treatment.

**Design** Population based cohort study using electronic health records from the Clinical Practice Research Datalink and Hospital Episode Statistics.

**Setting** UK primary care, 1997-2014.

**Participants** Patients starting treatment with angiotensin converting enzyme inhibitors or angiotensin receptor blockers (n=122 363).

**Main outcome measures** Poisson regression was used to compare rates of end stage renal disease, myocardial infarction, heart failure, and death among patients with creatinine increases of 30% or more after starting treatment against those without such increases, and for each 10% increase in creatinine. Analyses were adjusted for age, sex, calendar period, socioeconomic status, lifestyle factors, chronic kidney disease, diabetes, cardiovascular comorbidities, and use of other antihypertensive drugs and non-steroidal anti-inflammatory drugs.

**Results** Among the 2078 (1.7%) patients with creatinine increases of 30% or more, a higher proportion were female, were elderly, had cardiorenal comorbidity, and used non-steroidal anti-inflammatory drugs, loop diuretics, or potassium sparing diuretics. Creatinine increases of 30% or more were associated with an increased adjusted incidence rate ratio for all outcomes, compared with increases of less than 30%: 3.43 (95% confidence interval 2.40 to 4.91) for end stage renal disease, 1.46 (1.16 to 1.84) for myocardial infarction, 1.37 (1.14 to 1.65) for heart failure, and 1.84 (1.65 to 2.05) for death. The detailed categorisation of increases in creatinine concentrations (<10%, 10-19%, 20-29%, 30-39%, and ≥40%) showed a graduated relation for all outcomes (all P values for trends <0.001). Notably, creatinine increases of less than 30% were also associated with increased incidence rate ratios for all outcomes, including death (1.15 (1.09 to 1.22) for increases of 10-19% and 1.35 (1.23 to 1.49) for increases of 20-29%, using <10% as reference). Results were consistent across calendar periods, across subgroups of patients, and among continuing users.

**Conclusions** Increases in creatinine after the start of angiotensin converting enzyme inhibitor/angiotensin receptor blocker treatment were associated with adverse cardiorenal outcomes in a graduated relation, even below the guideline recommended threshold of a 30% increase for stopping treatment.

## Introduction

Angiotensin converting enzyme inhibitors (ACEI) and angiotensin receptor blockers (ARB) are commonly prescribed drugs for hypertension, heart failure, diabetic microalbuminuria, and proteinuric renal disease and after myocardial infarction.^1^ Patients may, however, have a sudden decline in kidney function after starting to take these drugs, owing to antagonism of angiotensin II mediated efferent arteriolar constriction.^2^ Despite unambiguous recommendations to detect sudden renal impairment by monitoring serum creatinine before and after the start of ACEI/ARB treatment and to discontinue treatment if creatinine concentrations increase by 30% or more,^1^ recent data show that only 10% of patients receive the recommended monitoring and only 20% of those with a creatinine increase of 30% or more after starting ACEI/ARB treatment discontinue the drugs.^3^

Clinical trial data has indicated that ACEI/ARB induced renal impairment is uncommon.^4^ ^5^ Patients seen in routine clinical practice are, however, on average older and have more comorbidity than those eligible for trials.^6^ As a consequence, the absolute risk of increases in creatinine of 30% or more in the community setting is not negligible.^3^ Although this level of creatinine increase after starting ACEI/ARB treatment raises concern about the long term balance of risks and benefits, smaller increases (<30%) do not prompt consideration of treatment discontinuation according to current guidelines. The rationale for the 30% threshold in the context of adverse clinical outcomes is unclear,^4^ as little evidence is available on the actual risks associated with creatinine increases of less than 30%.

Considering the high prevalence of ACEI/ARB use in general practice, any additional previously unrecognised risks would have major clinical and public health implications. We therefore used real world data to examine the cardiorenal risks associated with different levels of increase in creatinine after the start of ACEI/ARB treatment.

## Methods

### Data sources

We used the UK’s Clinical Practice Research Datalink (CPRD), linked to hospital record data from the Hospital Episode Statistics (HES) database. The CPRD database contains data from primary care electronic health records for 7% of the UK population (approximately 15 million patient lives, with about 8 million currently followed).^7^ Patients included in the CPRD are largely representative of the UK population in terms of age, sex, and ethnicity.^7^ ^8^ Information recorded in the database covers demographics such as sex and year of birth, the location of the general practice, medical diagnoses (based on Read codes), drug prescriptions, and a range of routine laboratory test results. The HES records all hospital admissions for patients covered by the National Health Service who receive treatment from either English NHS trusts or independent providers.^7^ ^8^ Fifty eight per cent of general practices included in the CPRD have agreed to HES linkage.^7^ We used lists of Read codes (CPRD) and ICD-10 (international classification of diseases, 10th revision) codes (HES) to identify outcomes and covariables. We obtained linked data on socioeconomic status based on area of residence from the UK Index of Multiple Deprivation.

### Study population

We identified a cohort of all HES linked CPRD patients aged 18 years or above who started ACEI/ARB treatment between 1 April 1997 and 31 March 2014. We defined new users as those with at least one year of continuous registration in the CPRD before their first recorded prescription for ACEI/ARB. We restricted our main study cohort to patients with both pre-initiation (within 12 months) and post-initiation (within two months) creatinine measurements and excluded patients with end stage renal disease diagnosed before cohort entry (n=17).

### Serum creatinine

We extracted all creatinine test results from the general practice records of the study population. We calculated a change in creatinine concentrations after the start of ACEI/ARB treatment as the relative difference between the most recent baseline measurement before or on the date of starting treatment and the first follow-up measurement within two months after starting. We defined the baseline measurement as within 12 months because previous work suggested that very recent creatinine concentrations are obtained for only a small proportion of patients starting ACEI/ARBs.^3^ We chose the two month post-initiation period to accord with the interval recommended in reviews of previous trial data.^4^

In our analysis, we firstly dichotomised the relative increase according to the guideline recommended cut-off levels of 30% or more versus less than 30%. Secondly, to examine whether a graduated (“dose-response”) relation existed, we categorised increases in creatinine in more detail, as less than 10% (reference group), 10-19%, 20-29%, 30-39%, and 40% or more. Thirdly, we used fractional polynomials to assess the form of the association between the continuous creatinine increase variable and outcomes. Because of evidence of non-linearity in the log scale for the association with several of the outcomes, we kept to the categorical modelling.

### Outcomes

We used HES and the CPRD to identify first time diagnoses of end stage renal disease, myocardial infarction, and heart failure, as well as all cause mortality. We defined end stage renal disease as the presence of a hospital or primary care morbidity code for end stage renal disease, renal transplant, peritoneal dialysis or haemodialysis, or an arteriovenous fistula (suggesting anticipation of end stage renal disease).

### Patients’ characteristics

We obtained information for all patients on age, sex, socioeconomic status (fifths of 2004 Index of Multiple Deprivation scores), lifestyle factors (smoking, alcohol intake, and body mass index), comorbidities (diabetes, myocardial infarction, heart failure, hypertension, arrhythmia, peripheral arterial disease, and chronic kidney disease stage), blood pressure measurements before and after starting ACEI/ARB treatment, and concomitant use of other antihypertensive drugs (β blockers, calcium channel blockers, thiazides, loop diuretics, and potassium sparing diuretics) and non-steroidal anti-inflammatory drugs at time of starting ACEI/ARB treatment.^9^ We used algorithms to estimate smoking status, alcohol intake, and body mass index based on the most recent CPRD records before the start of ACEI/ARB treatment.^10^ ^11^ We calculated estimated glomerular filtration rate on the basis of the baseline creatinine concentration and the chronic kidney disease stage by using the CKD-EPI equation.^12^

We identified other comorbidities from the CPRD and HES on the basis of diagnoses recorded before the start of ACEI/ARB treatment. We defined pre-initiation and post-initiation systolic and diastolic blood pressure on the basis of the most recent measurement within 12 months before and after the start of ACEI/ARB treatment. Use of non-steroidal anti-inflammatory drugs was based on prescriptions recorded within 30 days before the start of ACE/ARB treatment. We defined concurrent use of other antihypertensive drugs by courses of continuous treatment for each class of drugs concomitant with the ACEI/ARB prescription date. In identifying continuous courses of treatment, we calculated the end date of each prescription by adding the duration of the prescription (total number of tablets prescribed divided by the specified number of tablets per day) to the date of the prescription. We further allowed for a 30 day gap between the end date of one prescription and the start of the next consecutive prescription to allow for alternative sources of drug (eg, outpatient clinics) or stockpiling of prescriptions.

### Patient involvement

No patients were involved in setting the research question or the outcome measures, nor were they involved in developing plans for recruitment, design, or implementation of the study. No patients were asked to advise on interpretation or writing up of results. There are no plans to disseminate the results of the research to study participants or the relevant patient community.

### Statistical analysis

We characterised all patients starting ACEI/ARB treatment according to sex, age, comorbidities, co-medication use, socioeconomic status, lifestyle factors, and calendar period. We followed all new ACEI/ARB users with a change in creatinine concentration between baseline and the date of the first follow-up test, until the occurrence of an outcome, death, withdrawal from the general practice, or end of the follow-up period (31 March 2014), whichever occurred first. We illustrated the survival function by using the Kaplan-Meier estimator.

We used Poisson regression to examine the association between the percentage increase in creatinine concentration and long term cardiorenal risks. We modelled the cause specific hazard to account for competing risks (that is, censoring outcomes competing with the outcome of interest), which is appropriate for estimating causal effects.^13^ ^14^ We calculated rates and incidence rate ratios comparing the associations of categories of percentage creatinine increase with outcomes, using robust standard errors to account for clustering by general practice. We adjusted for age and sex in the “crude” model. In the main analysis, we also adjusted for the comorbidities listed above (including chronic kidney disease stage at baseline), use of concurrent drugs, lifestyle factors, socioeconomic status, calendar period, and time since first prescription. We included age (<50, 50-59, 60-69, 70-79, and ≥80 years), calendar period (1997-2003, 2004-08, and 2009-14) and years since first prescription (<1, 1 to <2, 2 to <5, 5 to <10, and ≥10 years) as time updated variables. To restrict assessment of outcomes to patients with incident disease, in each analysis we excluded people with a previous history (assessed at baseline) of the outcome in question. To examine whether patients’ characteristics modified the incidence rate ratios, we stratified the analyses by comorbidities. We also illustrated the time dependent effect estimates for each outcome graphically and did tests for linear trends to examine whether an interaction with time since starting drug treatment existed.

To consider the effect of potential confounders, we examined whether the effect estimates differed from our main results in several sensitivity analyses. Firstly, we restricted the study period to the most recent 10 year calendar period (2004-14) to increase the completeness of covariable recording and to take into account temporal differences in patient care.^15^ Secondly, we excluded patients with diabetes or chronic kidney disease stage 4 to account for measurements made at outpatient hospital clinics and therefore not available in the CPRD for these groups of patients. Thirdly, to explore the effect of drug cessation, we restricted the analysis to continuing users (irrespective of creatinine result)—that is, patients whose first continuous course of ACE/ARB treatment ended at least 90 days after the retest date. Fourthly, to consider the potential confounding effect of proteinuria, we restricted an analysis to ACEI/ARB users with diabetes, among whom we would anticipate that most have substantial protein excretion. Fifthly, we excluded patients with a potassium concentration above 6 mmol/L at the first follow-up monitoring to explore the prognostic influence of hyperkalaemia on the outcomes, particularly death. Sixthly, to gain insight into potential alternative mechanisms leading to increases in creatinine after the start of ACEI/ARB treatment, we added a post hoc analysis to estimate the relative reduction in median systolic and diastolic blood pressure after the start of treatment. Finally, we examined whether our cohort differed from other patients starting ACEI/ARB treatment who did not have both pre-initiation and post-initiation creatinine monitoring. For this purpose, we resampled all patients starting ACEI/ARB treatment in the study period to compare baseline characteristics and cumulative mortality risk among those with complete versus incomplete pre-initiation and post-initiation monitoring. We used the STATA 14 statistical software package for all analyses.

## Results

### Patients’ characteristics

Among 303 451 patients who started ACEI/ARB treatment during 1997-2014, 122 363 (40%) had both baseline and follow-up creatinine monitoring and were included in the study (table 1). Among these, 2078 (1.7%) had an increase in creatinine of 30% or more (median age 68 years) and 120 285 (98.3%) had an increase of less than 30% (median age 63 years). More detailed categorisation showed that the creatinine increase was less than 10% for 102 445 (83.7%) patients, 10-19% for 14 301 (11.7%) patients, 20-29% for 3539 (2.9%) patients, 30-39% for 1099 (0.9%) patients, and 40% or more for 979 (0.8%) patients.

**Table 1 tbl1:** Patients’ characteristics according to guideline recommended discontinuation level of creatinine increases (≥30%) after renin-angiotensin system blockade. Values are numbers (percentages) unless stated otherwise

Characteristic	Serum creatinine elevation after starting ACEI/ARB	
	≥30% (n=2078)	<30% (n=120 285)
Female sex	1166 (56.1)	55 482 (46.1)
Age, years:		
<50	292 (14.1)	21 959 (18.3)
50-59	322 (15.5)	27 955 (23.2)
60-69	452 (21.8)	31 820 (26.5)
70-79	540 (26.0)	25 908 (21.5)
≥80	472 (22.7)	12 643 (10.5)
Comorbidities^*^		
Diabetes mellitus	494 (23.8)	26 433 (22.0)
Myocardial infarction	219 (10.5)	5468 (4.5)
Heart failure	395 (19.0)	5756 (4.8)
Hypertension	1333 (64.1)	91 042 (75.7)
Arrhythmia	358 (17.2)	8122 (6.8)
Peripheral arterial disease	124 (6.0)	3044 (2.5)
Chronic kidney disease (eGFR)^†^:		
Stage ≤2 (**≥**60)	1612 (77.6)	98 702 (82.1)
Stage 3a (45-59)	281 (13.5)	16 387 (13.6)
Stage 3b (30-44)	143 (6.9)	4502 (3.7)
Stage 4 (15-29)	42 (2.0)	694 (0.6)
Co-medications		
β blockers	493 (23.7)	20 474 (17.0)
Calcium channel blockers	352 (16.9)	22 700 (18.9)
Thiazides	435 (20.9)	25 281 (21.0)
Loop diuretics	594 (28.6)	8693 (7.2)
Potassium sparing diuretics	183 (8.8)	2354 (2.0)
NSAIDs	706 (34.0)	28 306 (23.5)
Blood pressure, median (IQR)^‡^:		
Pre-initiation systolic	150 (135-168)	155 (142-169)
Pre-initiation diastolic	84 (75-95)	90 (80-98)
Post-initiation systolic	140 (125-158)	144 (132-158)
Post-initiation diastolic	80 (70-90)	83 (76-90)
Socioeconomic status, fifths:		
1 (lowest)	468 (22.5)	29 144 (24.2)
2	469 (22.6)	28 463 (23.7)
3	460 (22.1)	25 681 (21.4)
4	388 (18.7)	21 799 (18.1)
5 (highest)	287 (13.8)	15 040 (12.5)
Missing	6 (0.3)	158 (0.1)
Smoking status:		
Never	687 (33.1)	41 528 (34.5)
Ever	1373 (66.1)	78 574 (65.3)
Missing	18 (0.9)	183 (0.2)
Alcohol intake:		
No use	276 (13.3)	12 951 (10.8)
Current	1488 (71.6)	94 129 (78.3)
Former	162 (7.8)	8146 (6.8)
Missing	152 (7.3)	5059 (4.2)
Body mass index group:		
Underweight	47 (2.3)	1115 (0.9)
Healthy weight	560 (26.9)	28 676 (23.8)
Overweight	717 (34.5)	46 231 (38.4)
Obesity	603 (29.0)	40 116 (33.4)
Missing	151 (7.3)	4147 (3.4)
Calendar period:		
1997-2003	364 (17.5)	16 157 (13.4)
2004-08	983 (47.3)	59 915 (49.8)
2009-14	731 (35.2)	44 213 (36.8)

ACEI=angiotensin converting enzyme inhibitor; ARB=angiotensin receptor blocker; eGFR=estimated glomerular filtration rate; IQR=interquartile range; NSAID=non-steroidal anti-inflammatory drug.

*Diagnosis ever registered in Clinical Practice Research Datalink or Hospital Episode Statistics before start of treatment with ACEI or ARB.

†Calculated from most recent creatinine measurement within 12 months before first prescription date; eGFR given in mL/min/1.73 m^2^.

‡16 365 (13%) patients had no pre-initiation blood pressure measurement within 12 months before starting ACEI/ARB treatment (18% among those with ≥30% increase in creatinine and 13% among those with <30% increase). Also, 17 190 (14%) patients had no post-initiation blood pressure measurement in 12 months after starting drug treatment (19% among those with ≥30% increase in creatinine and 14% among those with <30% increase).

Compared with patients with a creatinine increase of less than 30%, a higher proportion of those with an increase of 30% or more were female (56.1% *v* 46.1%) or had moderate to severe chronic kidney disease (stage 3b or 4) (8.9% *v* 4.3%), previous myocardial infarction (10.5% *v* 4.5%), heart failure (19.0% *v* 4.8%), arrhythmia (17.2% *v* 6.8%), or peripheral arterial disease (6.0% *v* 2.5%). Patients with an increase of 30% or more were four times more likely to use loop diuretics (28.6% *v* 7.2%) or potassium sparing diuretics (8.8% *v* 2.0%) but also more often used β blockers (23.7% *v* 17.0%) and non-steroidal anti-inflammatory drugs (34.0% *v* 23.5%); fewer had hypertension (64.1% *v* 75.7%), calcium channel blocker use (16.9% *v* 18.9%), current alcohol consumption (71.6% *v* 78.3%), or obesity (29.0% *v* 33.4%). The overall blood pressure response was similar in the two groups after the start of ACEI/ARB treatment, both having a 7% reduction in systolic blood pressure (from 150 to 140 mm Hg in patients with a creatinine increase of 30% or more and from 155 to 144 mm Hg in those with a less than 30% increase). Socioeconomic status, use of thiazides, prevalence of smoking, and prevalence of diabetes did not differ between the groups.

### Levels of creatinine increase and clinical outcomes

Increases in creatinine of 30% or more were associated with increased rates of all outcomes (table 2). The adjusted incidence rate ratios were 3.43 (95% confidence interval 2.40 to 4.91) for end stage renal disease, 1.46 (1.16 to 1.84) for myocardial infarction, 1.37 (1.14 to 1.65) for heart failure, and 1.84 (1.65 to 2.05) for death.

**Table 2 tbl2:** Creatinine increases of ≥30% after renin-angiotensin system blockade and risk of adverse cardiorenal events^*^

Serum creatinine increase^†^	No of events	Risk, % (95% CI)^‡^			Rate per 1000 person years	Incidence rate ratio (95% CI)	
		1 year	5 years	10 years		Age and sex adjusted	Fully adjusted^§^
End stage renal disease:							
<30%	762	0.05 (0.04 to 0.07)	0.33 (0.29 to 0.37)	0.77 (0.68 to 0.86)	1.3	1.00 (reference)	1.00 (reference)
≥30%	45	0.30 (0.13 to 0.63)	0.74 (0.41 to 1.25)	1.92 (1.02 to 3.30)	5.2	4.06 (3.01 to 5.48)	3.43 (2.40 to 4.91)
Myocardial infarction:							
<30%	3334	0.41 (0.37 to 0.45)	1.75 (1.67 to 1.84)	3.68 (3.5 to 3.88)	5.9	1.00 (reference)	1.00 (reference)
≥30%	87	0.28 (0.11 to 0.64)	2.19 (1.51 to 3.07)	3.80 (2.69 to 5.19)	11.0	1.73 (1.41 to 2.13)	1.46 (1.16 to 1.84)
Heart failure:							
<30%	6892	0.95 (0.90 to 1.01)	3.22 (3.10 to 3.34)	7.28 (7.00 to 7.56)	12.4	1.00 (reference)	1.00 (reference)
≥30%	208	2.94 (2.19 to 3.85)	5.89 (4.73 to 7.23)	9.01 (7.17 to 11.1)	28.9	2.12 (1.82 to 2.47)	1.37 (1.14 to 1.65)
All cause mortality:							
<30%	13281	1.74 (1.67 to 1.82)	9.68 (9.48 to 9.88)	22.5 (22.1 to 23.0)	22.4	1.00 (reference)	1.00 (reference)
≥30%	640	11.1 (9.77 to 12.5)	29.8 (27.6 to 32.1)	49.2 (45.5 to 53.0)	72.7	2.68 (2.47 to 2.91)	1.84 (1.65 to 2.05)

*Among patients with at least one creatinine measurement within 12 months before and 2 months after starting drug and who continued treatment after first follow-up measurement.

†Increase calculated as difference between most recent baseline measurement within 12 months before starting drug and first follow-up measurement within 2 months after starting drug.

‡Cumulative incidence proportions of non-fatal outcomes calculated taking into account death as competing risk.

§Adjusted for age, sex, comorbidities (diabetes mellitus, myocardial infarction, heart failure, hypertension, arrhythmia, peripheral arterial disease, and chronic kidney disease stage), co-medications (β blockers, calcium channel blockers, thiazides, loop diuretics, potassium sparing diuretics, and non-steroidal anti-inflammatory drugs), lifestyle factors (smoking status, alcohol intake, and body mass index), socioeconomic status, calendar period, and time since first prescription.

When we examined interactions with time since the start of drug treatment (fig 1 and supplementary table A), we observed a pronounced effect of time for end stage renal disease, with increases in incidence rate ratios falling from 12.2-fold during the first year to 3.7-fold within the second year, to 1.7-fold within 2 to <5 years, and to 2.5-fold within 5 to <10 years after the start of treatment. However, confidence intervals were wide, reflecting the relatively small number of end stage renal disease events (P for trend=0.094). We observed similar trends of decreasing risk over time for heart failure (P for trend=0.025) and mortality (P for trend<0.001), although effect sizes were smaller. The incidence rate ratio for heart failure fell from a 1.9-fold increase within the first year to a 1.5-fold increase within the second year and remained neutral in risk thereafter. The mortality rate ratio declined from a 3.5-fold increase within the first year and remained approximately 50% increased thereafter.

**Figure f1:**
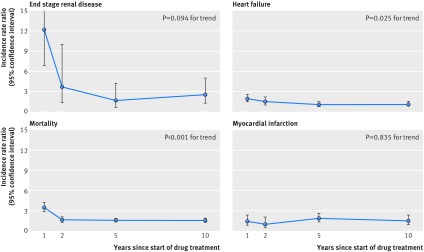
**Fig 1** Time dependent cardiorenal risks associated with creatinine increases ≥30% after renin-angiotensin system blockade

The more detailed categorisation of creatinine increases showed graduated effects for all outcomes. This is illustrated by the survival function in figure 2. The absolute one year risk of dying was 2% in the group with less than 10% increase, 2% for 10-19%, 4% for 20-29%, 7% for 30-39%, and 16% for 40% or above; the corresponding risks were 9%, 12%, 16%, 24%, and 37% at five years and 22%, 26%, 33%, 42%, and 57% at 10 years. This “dose-response” relation also held for all outcomes after adjustment for possible confounders (fig 3). Using creatinine increase less than 10% as reference, incidence rate ratios increased steadily among patients with creatinine increases of 10-19% up to those with creatinine increases of 40% or more for end stage renal disease (1.73 to 4.04; P for trend<0.001), for myocardial infarction (1.12 to 1.59; P<0.001), for heart failure (1.14 to 1.42; P<0.001), and for death (1.15 to 2.11; P<0.001).

**Figure f2:**
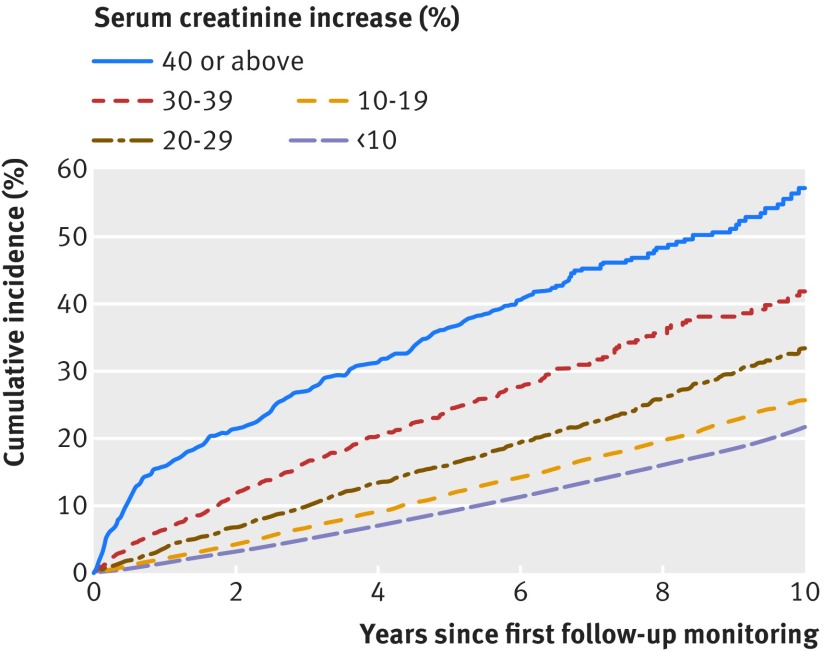
**Fig 2** Cumulative mortality according to levels of creatinine increase after renin-angiotensin system blockade

**Figure f3:**
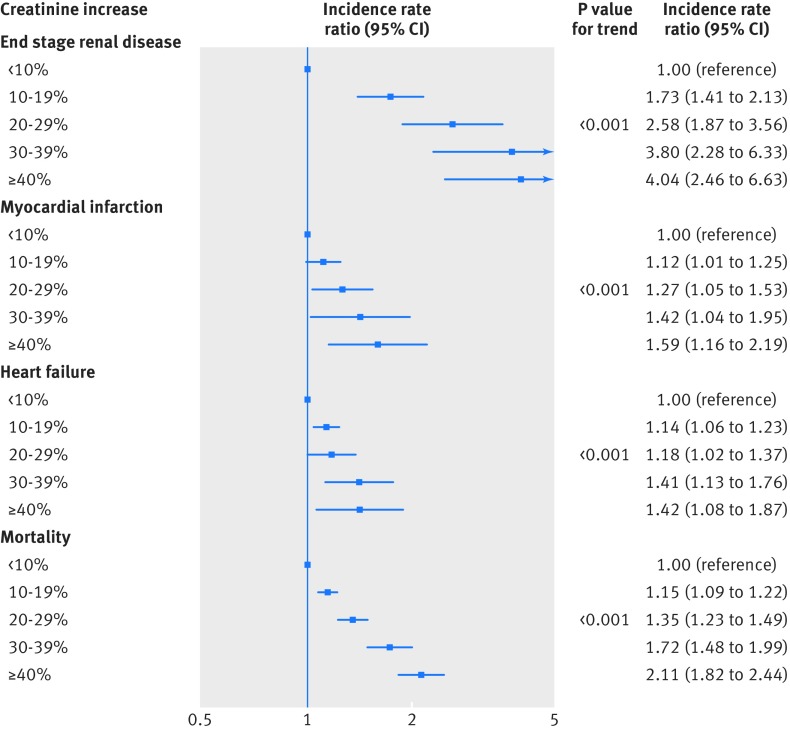
**Fig 3** Cardiorenal risks associated with levels of creatinine increase after renin-angiotensin system blockade

### Patient subgroups

Among subgroups of patients (table 3), the risk of adverse renal or cardiac outcomes associated with creatinine increases of 30% or more was higher in men than in women. The precision of estimates for non-fatal outcomes varied by subgroups, but without substantial modification of the incidence rate ratios. Importantly, the incidence rate ratio for death had high precision for all subgroups and was consistently increased in patients with and without individual comorbidities, including diabetes.

**Table 3 tbl3:** Creatinine increases ≥30% after renin-angiotensin system blockade and risk of adverse cardiorenal events, stratified by comorbidities

Baseline characteristics	Adjusted incidence rate ratio (95% CI)			
	End stage renal disease	Myocardial infarction	Heart failure	All cause death
Sex:				
Men	4.81 (3.22 to 7.21)	1.64 (1.24 to 2.17)	1.51 (1.20 to 1.91)	1.89 (1.62 to 2.20)
Women	1.64 (0.75 to 3.58)	1.30 (0.89 to 1.88)	1.25 (0.94 to 1.65)	1.74 (1.50 to 2.03)
Diabetes mellitus	3.19 (1.81 to 5.61)	1.82 (1.28 to 2.60)	1.32 (0.95 to 1.85)	1.96 (1.66 to 2.32)
No diabetes mellitus	3.09 (1.91 to 5.01)	1.31 (0.97 to 1.78)	1.40 (1.13 to 1.73)	1.78 (1.55 to 2.04)
Previous myocardial infarction	1.12 (0.21 to 6.00)	-	1.34 (0.85 to 2.10)	1.93 (1.53 to 2.43)
No myocardial infarction	3.62 (2.50 to 5.24)	-	1.42 (1.16 to 1.75)	1.84 (1.63 to 2.06)
Heart failure	1.86 (0.40 to 8.74)	1.63 (0.96 to 2.78)	-	1.85 (1.54 to 2.23)
No heart failure	3.86 (2.70 to 5.53)	1.47 (1.12 to 1.91)	-	1.85 (1.63 to 2.10)
Hypertension	4.53 (2.99 to 6.87)	1.65 (1.22 to 2.22)	1.61 (1.30 to 1.99)	1.94 (1.69 to 2.22)
No hypertension	1.92 (0.93 to 3.97)	1.21 (0.80 to 1.84)	1.14 (0.85 to 1.51)	1.76 (1.50 to 2.07)
Cardiac arrhythmia	3.83 (1.36 to 10.8)	1.70 (0.98 to 2.94)	1.35 (0.94 to 1.93)	1.68 (1.38 to 2.04)
No cardiac arrhythmia	3.49 (2.41 to 5.05)	1.44 (1.11 to 1.87)	1.42 (1.17 to 1.74)	1.93 (1.71 to 2.19)
Peripheral arterial disease	1.03 (0.14 to 7.67)	1.59 (0.83 to 3.06)	1.78 (1.06 to 2.98)	1.86 (1.32 to 2.61)
No peripheral artery disease	3.67 (2.58 to 5.22)	1.48 (1.16 to 1.88)	1.35 (1.10 to 1.64)	1.86 (1.68 to 2.06)
Chronic kidney disease (eGFR^*^):				
Stage ≤2 (**≥**60)	2.70 (1.61 to 4.50)	1.42 (1.06 to 1.89)	1.23 (0.99 to 1.53)	1.71 (1.49 to 1.96)
Stage 3a (45-59)	5.81 (2.82 to 12.0)	2.10 (1.33 to 3.31)	1.90 (1.30 to 2.77)	2.05 (1.62 to 2.60)
Stage 3b (30-44)	2.79 (1.06 to 7.34)	1.31 (0.54 to 3.17)	1.64 (0.96 to 2.81)	2.01 (1.45 to 2.77)
Stage 4 (15-29)	7.81 (1.99 to 30.7)	0.84 (0.09 to 7.94)	0.68 (0.09 to 5.18)	2.36 (1.28 to 4.37)

See table 2 and text for definitions of study cohort, serum creatinine increases, and adjusted model.

*Estimated glomerular filtration rate (mL/min/1.73 m^2^).

### Sensitivity analyses

The sensitivity analysis comparing the baseline characteristics of patients with and without complete monitoring of creatinine concentrations showed no major differences in age, sex, blood pressure values, socioeconomic status, or lifestyle factors (supplementary table B). However, those with complete monitoring had a higher prevalence of non-cardiac comorbidity, in particular diabetes and chronic kidney disease. The cumulative mortality function for this group was similar to that of the group with creatinine increases between 10% and 19% (supplementary figure A). The remaining sensitivity analyses all supported the robustness of the main results (supplementary tables C and D).

## Discussion

We found that patients in routine clinical care who started treatment with ACEI/ARB and whose creatinine concentration had increased by 30% or more at their first follow-up monitoring visit were at increased risk for adverse cardiac outcomes and death, compared with patients with more stable creatinine values. Our study thus confirms data from clinical trials in a real world clinical setting. Moreover, we established that risks were also substantially increased for end stage renal disease. In general, risks were highest in the first year after the start of ACEI/ARB treatment but were sustained up to 10 years later for end stage renal disease, myocardial infarction, and death. Importantly, we showed a “dose-response” relation between the level of increase in creatinine values and risk of adverse outcomes, indicating that all increases below 30% cannot be viewed as safe. Our results were consistent across calendar periods and patient subgroups in a range of sensitivity analyses. It is not clear whether increases in creatinine values after the start of ACEI/ARB treatment are due to pathophysiological processes representing a biomarker of increased risk or whether a direct causal relation exists between reduced renal function and adverse outcomes. These results therefore identify a group of patients at high risk but do not necessarily support discontinuation of ACEI/ARBs.

### Strengths and limitations of study

This large population based study is the first to use data from routine clinical care to examine long term outcomes associated with changes in renal function after the start of ACEI/ARB treatment. It represents an important complement to clinical trials, the participants of which may not be representative of treated patients in clinical practice.^6^ The study’s size and long follow-up also permitted examination of a full range of outcomes, beyond those evaluated in individual clinical trials. Importantly, this is the first study to examine the association with end stage renal disease, as clinical trials are rarely powered to examine this outcome.

Patients who had a greater fall in renal function after starting ACEI/ARB treatment had a higher proportion of comorbidities and concurrent drugs that are themselves associated with adverse renal outcomes. However, our findings were robust after adjustment for a range of potential confounders, including comorbidity, co-medication use, lifestyle factors, and socioeconomic status. Nevertheless, residual confounding cannot be excluded. We were unable to adjust for proteinuria, a potentially important confounder owing to its association with adverse cardiorenal outcomes, because of its incomplete recording. However, to provide an explanation for our results, proteinuria would need to be associated with the degree of increase in creatinine concentrations after the start of ACEI/ARB treatment. We are not aware of any evidence that this is the case. In addition, effect estimates were similar in all analyses restricted to patients with diabetes, among whom we would anticipate that a high proportion would have substantial urinary protein excretion.

The validity of the diagnosis of myocardial infarction has consistently been found to be high, with positive predictive values of 92-93% in both the CPRD and HES.^16^ ^17^ Heart failure, end stage renal disease, and mortality have not been validated individually. However, the diagnoses recorded in the CPRD, particularly in the domains assessed by the Quality and Outcomes Framework,^18^ ^19^ are in general considered to have adequate validity for research purposes, with an overall median proportion of cases with a confirmed diagnosis of 89%.^20^ ^21^

A limitation of our study was that we could include only patients with both baseline and follow-up creatinine measurements (complete case analysis) to calculate changes in renal function. Comparison of the baseline characteristics of patients with and without complete monitoring of creatinine concentrations showed no major differences in demographics, socioeconomics, or lifestyle, although a greater proportion of those with complete monitoring had diabetes and chronic kidney disease. Therefore, the proportion of patients with a decline in renal function among those starting ACEI/ARB treatment in the population as a whole may be lower than that observed in the monitored group. This view was also supported by the cumulative mortality function in the group with incomplete monitoring, which was similar to the monitored group with less pronounced increases in creatinine. Importantly, we have no reason to suspect that the association between change in renal function and long term outcomes is not generalisable to the whole population. Also, our results were consistent within strata of patients’ comorbidities and when we excluded subgroups of patients expected to have monitoring performed in outpatient hospital clinics.

Although we used the most recent blood test within 12 months, two thirds of all baseline creatinine tests were carried out within six months of the start of ACE/ARB treatment. Our study was also able to focus on participants whom we were confident continued to be prescribed ACEI/ARBs after their post-initiation blood test (regardless of creatinine results). We previously found that 80% of patients with creatinine increases of 30% or more continued treatment despite guideline recommendations to stop.^3^ Our new results emphasise the clinical implications of these findings, as the adverse outcomes associated with creatinine increases also applies to continuing ACEI/ARB users.

General practice system software used for issuing prescriptions ensures the accuracy of prescription data, but we cannot be certain that patients were taking their drugs as prescribed. However, given the consistency of results for the overall cohort and for patients with prescription coverage 90 days after the monitoring date, misclassified drug use is unlikely to have affected the results substantially.

### Comparison with other studies

Many post hoc analyses of clinical trials have examined the prognostic significance of a deterioration in renal function after the start of ACEI/ARB treatment. In clinical trials of patients with heart failure, deterioration in renal function after starting ACEI/ARB treatment is commonly found.^22^ Although this deterioration is associated with a poorer prognosis compared with patients with preserved renal function, the overall benefits of ACEI/ARB treatment compared with placebo remain for cardiovascular outcomes and mortality.^22^ Our study does not undermine that evidence but flags that the risk-benefit ratio may differ among patients with marked changes in creatinine concentrations. This is particularly the case for other prescribing indications for which the clinical trial evidence is less clear.

The recommendation in many international guidelines to stop ACEI/ARB treatment if creatinine rises by 30% or more after initiation are founded on a single review of 12 clinical trials of ACEI/ARB treatment for diabetes and heart failure.^4^ Studies included in this review evaluated progression of renal disease among patients with pre-existing renal impairment. Of these studies, only six were double blinded and included a total of 1102 participants. These trials were published during 1993-97 and may not relate to patients receiving contemporary routine clinical care. The methods that define a cut-off level of creatinine increase at 30% for cessation are not clearly presented.^4^ In addition, the results provided by these studies are not supported by later trials. Recent reviews have not shown the superiority of ACEI/ARBs compared with other antihypertensive drugs for treating early non-diabetic chronic kidney disease,^23^ diabetes with normal renal function,^24^ and diabetes and chronic kidney disease.^25^ A UK multicentre interventional trial to compare the outcomes of continuation versus cessation of ACEI/ARB treatment is under way in response to observational evidence that stopping ACEI/ARB treatment may slow progression in advanced renal disease.^26^ ^27^

A fixed recommendation to stop ACEI/ARB treatment only if creatinine is increased by 30% or more is also hard to reconcile with the growing body of evidence related to acute kidney injury, which shows that even a small deterioration in renal function is associated with a subsequently increased risk of mortality and other adverse outcomes.^28^ It is important to consider that the prognostic significance of ACEI/ARB associated renal impairment may depend on the underlying cause and on subsequent changes in renal function if ACEI/ARB treatment is continued.^4^ ^22^ Underlying causes may be different in the routine care setting, in which patients are older, have multiple comorbidities, and have more advanced kidney disease compared with patients who participated in early clinical trials.

### Conclusions and implications

In routine primary care, most patients starting treatment with an ACEI/ARB have only minor changes in renal function. However, increases in creatinine concentrations of more than 10% after starting ACEI/ARB treatment affect more than 15% of patients and have important implications. We have shown that creatinine increases after the start of ACEI/ARB treatment were associated with cardiorenal risks in a “dose-response” relation, with no distinct cut-off at 30%, as previously suggested. Further investigation is needed to ascertain whether ACEI/ARB associated changes in renal function unmask underlying pathophysiology or lead directly to adverse outcomes by causing permanent renal impairment in some patients. In addition, a better understanding of the overall risk-benefit ratio of continuing treatment after loss of kidney function for different prescribing indications is needed. Most importantly, patients with substantial increases in creatinine after starting ACEI/ARB treatment should be recognised as a very high risk group needing close ongoing monitoring. Review is needed of the risks and potential benefits of continuation of drug treatment for the specific prescribing indication for each patient.

**What is already known on this topic**

A sudden decline in kidney function may occur after treatment with angiotensin converting enzyme inhibitors (ACEI) and angiotensin receptor blockers (ARB) is started
Increases in creatinine of up to 30% over baseline levels are generally considered safe and even a marker of long term preservation of kidney function
Long term cardiac and renal outcomes associated with more detailed categorisations of post-initiation increases in creatinine concentrations are unknown

**What this study adds**

This cohort study shows a graduated increased risk of end stage renal disease, adverse cardiac outcomes, and death for each 10% increase in creatinine, even below the 30% threshold
Whether these creatinine changes are causally related to adverse outcomes or represent a biomarker of increased risk is unclear
Increases in creatinine after starting ACEI/ARB treatment identify a high risk group needing close monitoring and in whom the risks and benefits of ACEI/ARB prescribing should be considered

## Web Extra

Extra material supplied by the author

## Notes

Contributors: LAT had the idea for the study and acquired data permissions. MS, KEM, and LAT designed the study. MS and KEM managed the data and established the cohort. MS, KEM, and LAT reviewed the literature. MS did the analyses. All authors participated in the discussion and interpretation of the results. MS organised the writing and wrote the initial drafts. All authors critically revised the manuscript for intellectual content and approved the final version. MS is the guarantor.

Ethical approval: The study protocol was approved by the London School of Hygiene and Tropical Medicine Ethics Committee (No 6536) and the Independent Scientific Advisory Committee (ISAC) for Medicines and Healthcare Products Regulatory Agency (No 16_025) and made available to the journal reviewers.

Transparency declaration: The lead author affirms that the manuscript is an honest, accurate, and transparent account of the study being reported; that no important aspects of the study have been omitted; and that any discrepancies from the study as planned have been explained.

Data sharing: No additional data available.
