# Supplementary material for: Serum creatinine elevation after renin-angiotensin system blockade and long term cardiorenal risks: cohort study
Source: BMJ. 2017 Mar 9;356:j791. doi: 10.1136/bmj.j791 (PMC5421447; doi:10.1136/bmj.j791)

Supplemental online material

Supplementary table A. Time-dependent cardiorenal risks associated with creatinine increases ≥30% after renin-angiotensin system blockade

Supplementary table B. Characteristics of patients initiating angiotensin converting-enzyme inhibitors or angiotensin-receptor blockers in the UK primary care during 1997-2014, by monitoring status

Supplementary table C. Creatinine increases ≥30% after renin-angiotensin system blockade and risk of adverse cardio-renal events: sensitivity analyses (1) restricted to 2004-2014; (2) excluding patients with diabetes/stage 4 chronic kidney disease; (3) restricted to continuing users; (4) restricted to diabetic users; and (5) excluding patients with hyperkalaemia*

Supplementary table D. Cardiorenal risks associated with detailed levels of creatinine increases following renin-angiotensin system blockade: sensitivity analyses (1) restricted to 2004-2014; (2) excluding patients with diabetes/stage 4 chronic kidney disease; (3) restricted to continuing users; (4) restricted to diabetic users; and (5) excluding patients with hyperkalaemia*

Supplementary figure A. Cumulative mortality of incompletely monitored patients and according to detailed levels of creatinine increases following renin-angiotensin system blockade

Supplementary table A. Time-dependent cardiorenal risks associated with creatinine increases ≥30% after renin-angiotensin system blockade*

	**Incidence rate ratio (95% CI)†**				**p-value for trend**
**Cardiorenal risks**	**<1 year**	**1 to <2 years**	**2 to <5 years**	**5 to <10 years**	
End stage renal disease	12.2 (6.9 to 21.7)	3.7 (1.3 to 10.3)	1.7 (0.6 to 4.2)	2.5 (1.2 to 5.0)	P=0.094
Myocardial infarction	1.4 (0.8 to 2.3)	0.9 (0.4 to 2.0)	1.8 (1.3 to 2.6)	1.5 (0.9 to 2.3)	P=0.835
Heart failure	1.9 (1.5 to 2.5)	1.5 (1.0 to 2.2)	1.1 (0.8 to 1.5)	1.0 (0.7 to 1.5)	P=0.025
All cause mortality	3.5 (2.9 to 4.2)	1.6 (1.3 to 2.1)	1.6 (1.3 to 1.8)	1.5 (1.2 to 1.9)	P<0.001

*Among patients with at least one creatinine measurement within 12 months before and 2 months after starting drug and who continued treatment after first follow-up measurement.

†Adjusted for age, sex, comorbidities (diabetes mellitus, myocardial infarction, heart failure, hypertension, arrhythmia, peripheral arterial disease, and chronic kidney disease stage), co-medications (β blockers, calcium channel blockers, thiazides, loop diuretics, potassium sparing diuretics, and non-steroidal anti-inflammatory drugs), lifestyle factors (smoking status, alcohol intake, and body mass index), socioeconomic status, calendar period, and time since first prescription.

Supplementary table B. Characteristics of patients initiating angiotensin converting-enzyme inhibitors or angiotensin-receptor blockers in the UK primary care sector during 1997-2014, by monitoring status

	**Pre-and post-initiation creatinine monitoring**		**Total**
	**Complete**	**Incomplete**	
**Total number**	122,363 (100)	181,088 (100)	303,451 (100)
**Female sex**	56,648 (46.3)	79,544 (43.9)	136,192 (44.9)
**Age (years)**			
<50 years	22,251 (18.2)	37,641 (20.8)	59,892 (19.7)
50-59 years	28,277 (23.1)	44,087 (24.3)	72,364 (23.8)
60-69 years	32,272 (26.4)	43,928 (24.3)	76,200 (25.1)
70-79 years	26,448 (21.6)	35,722 (19.7)	62,170 (20.5)
80+ years	13,115 (10.7)	19,710 (10.9)	32,825 (10.8)
**Comorbidities***			
Diabetes mellitus	26,927 (22.0)	29,935 (16.5)	56,862 (18.7)
Myocardial infarction	5,687 (4.6)	19,277 (10.6)	24,964 (8.2)
Heart failure	6,151 (5.0)	13,218 (7.3)	19,369 (6.4)
Hypertension	92,375 (75.5)	120,378 (66.5)	212,753 (70.1)
Arrhythmia	8,480 (6.9)	14,750 (8.1)	23,230 (7.7)
Peripheral arterial disease	3,168 (2.6)	4,735 (2.6)	7,903 (2.6)
Chronic kidney disease (eGFR)†			
Stage ≤2 (≥60)	100,314 (82.0)	104,294 (57.6)	204,608 (67.4)
Stage 3a (45–59)	16,668 (13.6)	14,433 (8.0)	31,101 (10.2)
Stage 3b (30–44)	4,645 (3.8)	3,425 (1.9)	8,070 (2.7)
Stage 4 (15–29)	736 (0.6)	545 (0.3)	1,281 (0.4)
Missing	0 (0)	58391 (32.2)	58391 (19.2)
**Comedications**			
Beta-blockers	20,967 (17.1)	39,908 (22.0)	60,875 (20.1)
Calcium channel blockers	23,052 (18.8)	24,642 (13.6)	47,694 (15.7)
Thiazides	25,716 (21.0)	33,146 (18.3)	58,862 (19.4)
Loop diuretics	9,287 (7.6)	18,234 (10.1)	27,521 (9.1)
Potassium sparing diuretics	2,537 (2.1)	4,638 (2.6)	7,175 (2.4)
NSAIDs	29,012 (23.7)	49,425 (27.3)	78,437 (25.8)
**Blood pressure, median (IQR)**			
Pre-systolic	155 (142 to 169)	156 (140 to 170)	156 (140 to 170)
Pre-diastolic	90 (80 to 98)	90 (80 to 100)	90 (80 to 100)
Post-systolic	144 (132 to 158)	144 (130 to 160)	144 (131 to 160)
Post-diastolic	83 (76 to 90)	84 (77 to 90)	83 (77 to 90)
**SES quintiles**			
1 (low)	29,612 (24.2)	43,762 (24.2)	73,374 (24.2)
2	28,932 (23.6)	41,780 (23.1)	70,712 (23.3)
3	26,141 (21.4)	37,643 (20.8)	63,784 (21.0)
4	22,187 (18.1)	33,963 (18.8)	56,150 (18.5)
5 (high)	15,327 (12.5)	23,717 (13.1)	39,044 (12.9)
Missing	164 (0.1)	223 (0.1)	387 (0.1)
**Smoking status**			

Never	42,215 (34.5)	66,912 (36.9)	109,127 (36.0)
Ever	79,947 (65.3)	112,344 (62.0)	192,291 (63.4)
Missing	201 (0.2)	1,832 (1.0)	2,033 (0.7)
**Alcohol intake**			
No use	13,227 (10.8)	22,680 (12.5)	35,907 (11.8)
Current	95,617 (78.1)	134,910 (74.5)	230,527 (76.0)
Former	8,308 (6.8)	10,202 (5.6)	18,510 (6.1)
Missing	5,211 (4.3)	13,296 (7.3)	18,507 (6.1)
**BMI groups**			
Underweight	1,162 (0.9)	1,955 (1.1)	3,117 (1.0)
Healthy weight	29,236 (23.9)	45,805 (25.3)	75,041 (24.7)
Overweight	46,948 (38.4)	67,513 (37.3)	114,461 (37.7)
Obesity	40,719 (33.3)	53,983 (29.8)	94,702 (31.2)
Missing	4,298 (3.5)	11,832 (6.5)	16,130 (5.3)
**Calendar period**			
1997-2003	16,521 (13.5)	63,156 (34.9)	79,677 (26.3)
2004-2008	60,898 (49.8)	75,276 (41.6)	136,174 (44.9)
2009-2014	44,944 (36.7)	42,656 (23.6)	87,600 (28.9)

Abbreviations: IQR, interquartile range; NSAIDs, nonsteroidal anti-inflammatory drugs

*Diagnosis ever registered in the CRPD or HES before initiation of treatment with angiotensin converting-enzyme inhibitors or angiotensin-receptor blockers.

† Calculated from most recent creatinine measurement within 12 months before the first prescription date. The parenthesis provides eGFR in ml/min/1.73m^2

Supplementary table C. Creatinine increases ≥30% after renin-angiotensin system blockade and risk of adverse cardio-renal events: sensitivity analyses (1) restricted to 2004-2014; (2) excluding patients with diabetes/stage 4 chronic kidney disease; (3) restricted to continuing users; (4) restricted to diabetic users; and (5) excluding patients with hyperkalaemia*

Serum creatinine increase†	Number of events	Rate per 1,000 person-years	Incidence rate ratio (95% confidence interval)	
			Age- and sex-adjusted	Fully adjusted‡
**1: 2004-2014**				
**End-stage renal disease**				
< 30%	531	1.2	1.00 (reference)	1.00 (reference)
≥ 30%	29	4.5	3.92 (2.67 to 5.75)	3.51 (2.25 to 5.49)
**Myocardial infarction**				
< 30%	2,392	5.5	1.00 (reference)	1.00 (reference)
≥ 30%	60	10.3	1.72 (1.35 to 2.21)	1.42 (1.09 to 1.86)
**Heart failure**				
< 30%	4,923	11.3	1.00 (reference)	1.00 (reference)
≥ 30%	151	28.3	2.20 (1.86 to 2.61)	1.47 (1.18 to 1.84)
**All-cause mortality**				
< 30%	9,025	19.7	1.00 (reference)	1.00 (reference)
≥ 30%	462	71.8	2.89 (2.63 to 3.17)	1.99 (1.76 to 2.25)
**2: Diabetes/CKD stage 4 excluded**				
**End-stage renal disease**				
< 30%	393	0.9	1.00 (reference)	1.00 (reference)
≥ 30%	23	3.6	4.08 (2.64 to 6.30)	3.25 (1.96 to 5.38)
**Myocardial infarction**				
< 30%	2,266	5.3	1.00 (reference)	1.00 (reference)
≥ 30%	52	9.0	1.56 (1.19 to 2.05)	1.33 (0.98 to 1.80)
**Heart failure**				
< 30%	4,841	11.4	1.00 (reference)	1.00 (reference)
≥ 30%	151	28.5	2.23 (1.88 to 2.65)	1.40 (1.13 to 1.73)
**All-cause mortality**				
< 30%	9,142	20.1	1.00 (reference)	1.00 (reference)
≥ 30%	444	68.3	2.71 (2.46 to 2.98)	1.80 (1.58 to 2.05)
**3: Continuing users§**				
**End-stage renal disease**				
< 30%	580	1.2	1.00 (reference)	1.00 (reference)
≥ 30%	25	4.0	3.50 (2.33 to 5.26)	3.26 (1.97 to 5.40)
**Myocardial infarction**				
< 30%	2,597	5.5	1.00 (reference)	1.00 (reference)
≥ 30%	55	9.9	1.74 (1.31 to 2.30)	1.45 (1.06 to 1.98)
**Heart failure**				
< 30%	5,395	11.5	1.00 (reference)	1.00 (reference)
≥ 30%	136	26.3	2.19 (1.81 to 2.66)	1.39 (1.11 to 1.75)
**All-cause mortality**				
< 30%	10,084	20.2	1.00 (reference)	1.00 (reference)
≥ 30%	321	51.4	2.22 (2.01 to 2.44)	1.53 (1.33 to 1.75)
**4: Diabetic users**				
**End-stage renal disease**				
< 30%	285	2.1	1.00 (reference)	1.00 (reference)
≥ 30%	17	7.8	3.77 (2.37 to 5.98)	3.19 (1.81 to 5.62)
**Myocardial infarction**				
< 30%	1,034	7.9	1.00 (reference)	1.00 (reference)
≥ 30%	33	16.3	2.03 (1.45 to 2.84)	1.81 (1.27 to 2.59)
**Heart failure**				
< 30%	1,982	15.4	1.00 (reference)	1.00 (reference)
≥ 30%	55	29.7	1.89 (1.43 to 2.50)	1.32 (0.95 to 1.85)
**All-cause mortality**				

< 30%	3,933	28.6	1.00 (reference)	1.00 (reference)
≥ 30%	168	76.0	2.46 (2.09 to 2.90)	1.96 (1.66 to 2.31)
**5: Hyperkalaemia excluded‖**				
**End-stage renal disease**				
< 30%	748	1.3	1.00 (reference)	1.00 (reference)
≥ 30%	43	5.0	3.99 (2.91 to 5.46)	3.30 (2.26 to 4.83)
**Myocardial infarction**				
< 30%	3,328	5.9	1.00 (reference)	1.00 (reference)
≥ 30%	86	11.0	1.73 (1.40 to 2.14)	1.46 (1.16 to 1.84)
**Heart failure**				
< 30%	6,867	12.4	1.00 (reference)	1.00 (reference)
≥ 30%	203	28.4	2.11 (1.81 to 2.45)	1.37 (1.13 to 1.65)
**All-cause mortality**				
< 30%	13,225	22.3	1.00 (reference)	1.00 (reference)
≥ 30%	620	71.4	2.65 (2.44 to 2.88)	1.84 (1.64 to 2.05)

* Among patients with at least one creatinine measurement within 12 months before and 2 months after drug initiation, who continued treatment after the first follow-up measurement (*i.e.*, the first continuous course of therapy ended at least 30 days after the first monitoring date, except in Sensitivity Analysis 3, in which a 90-day window was used)

[†] Increase calculated as the difference between the most recent baseline measurement within 12 months before drug initiation and the first follow-up measurement within two months after drug initiation.

[‡] Adjusted for age, sex, comorbidities (diabetes mellitus, myocardial infarction, heart failure, hypertension, arrhythmia, peripheral arterial disease, and chronic kidney disease stage), comedications (beta-blockers, calcium channel blockers, thiazides, loop diuretics, potassium sparing diuretics, and nonsteroidal anti-inflammatory drugs), lifestyle factors (smoking status, alcohol intake, and body mass index), socioeconomic status, calendar period, and time since first prescription.

[§] Prescription coverage > 90 days after retest

[‖] Potassium > 6 mmol/L

Supplementary table D. Cardiorenal risks associated with detailed levels of creatinine increases following renin-angiotensin system blockade: sensitivity analyses (1) restricted to 2004-2014; (2) excluding patients with diabetes/stage 4 chronic kidney disease; (3) restricted to continuing users; (4) restricted to diabetic users; and (5) excluding patients with hyperkalaemia*

	**Adjusted incidence rate ratio (95% confidence intervals)**			
**Creatinine increases**	**End-stage renal disease**	**Myocardial infarction**	**Heart failure**	**All-cause death**
**1: 2004-2014**				
<10%	1.00 (reference)	1.00 (reference)	1.00 (reference)	1.00 (reference)
10-19%	1.79 (1.42 to 2.27)	1.08 (0.95 to 1.23)	1.11 (1.01 to 1.22)	1.17 (1.09 to 1.26)
20-29%	2.53 (1.70 to 3.74)	1.17 (0.93 to 1.47)	1.15 (0.97 to 1.37)	1.37 (1.22 to 1.54)
30-39%	5.04 (2.88 to 8.81)	1.53 (1.09 to 2.16)	1.57 (1.22 to 2.02)	1.94 (1.66 to 2.27)
≥40%	2.94 (1.51 to 5.74)	1.36 (0.91 to 2.05)	1.45 (1.04 to 2.00)	2.20 (1.83 to 2.63)
**2: Diabetes/CKD stage 4 excluded**				
<10%	1.00 (reference)	1.00 (reference)	1.00 (reference)	1.00 (reference)
10-19%	1.59 (1.21 to 2.10)	1.07 (0.93 to 1.22)	1.14 (1.05 to 1.24)	1.14 (1.06 to 1.22)
20-29%	2.00 (1.20 to 3.34)	1.21 (0.94 to 1.55)	1.17 (0.99 to 1.37)	1.29 (1.16 to 1.45)
30-39%	3.24 (1.51 to 6.97)	1.24 (0.82 to 1.88)	1.47 (1.12 to 1.92)	1.76 (1.49 to 2.08)
≥40%	3.91 (2.07 to 7.38)	1.47 (0.96 to 2.25)	1.41 (1.04 to 1.91)	1.96 (1.60 to 2.40)
**3: Continuing users***				
<10%	1.00 (reference)	1.00 (reference)	1.00 (reference)	1.00 (reference)
10-19%	1.61 (1.26 to 2.06)	1.06 (0.93 to 1.20)	1.09 (1.01 to 1.19)	1.14 (1.07 to 1.22)
20-29%	2.72 (1.85 to 4.00)	1.08 (0.86 to 1.35)	1.17 (0.99 to 1.37)	1.30 (1.15 to 1.46)
30-39%	4.81 (2.68 to 8.61)	1.40 (0.94 to 2.09)	1.42 (1.07 to 1.87)	1.59 (1.32 to 1.92)
≥40%	1.95 (0.71 to 5.37)	1.55 (1.00 to 2.41)	1.43 (1.00 to 2.03)	1.57 (1.26 to 1.95)
**4: Diabetic users**				
<10%	1.00 (reference)	1.00 (reference)	1.00 (reference)	1.00 (reference)
10-19%	1.70 (1.22 to 2.36)	1.24 (1.03 to 1.50)	1.16 (1.00 to 1.35)	1.17 (1.06 to 1.29)
20-29%	2.43 (1.37 to 4.29)	1.42 (1.02 to 1.99)	1.26 (0.98 to 1.64)	1.52 (1.30 to 1.77)
30-39%	3.73 (1.80 to 7.71)	1.84 (1.12 to 3.05)	1.33 (0.87 to 2.04)	1.77 (1.40 to 2.24)
≥40%	3.39 (1.28 to 9.00)	1.98 (1.15 to 3.40)	1.42 (0.86 to 2.35)	2.37 (1.88 to 3.00)
**5: Hyperkalaemia excluded†**				
<10%	1.00 (reference)	1.00 (reference)	1.00 (reference)	1.00 (reference)
10-19%	1.75 (1.42 to 2.14)	1.12 (1.00 to 1.25)	1.14 (1.06 to 1.23)	1.15 (1.08 to 1.22)
20-29%	2.54 (1.83 to 3.51)	1.26 (1.04 to 1.53)	1.16 (1.01 to 1.34)	1.35 (1.22 to 1.48)
30-39%	3.82 (2.29 to 6.37)	1.39 (1.01 to 1.91)	1.39 (1.11 to 1.74)	1.69 (1.45 to 1.97)
≥40%	3.71 (2.15 to 6.43)	1.64 (1.20 to 2.25)	1.43 (1.07 to 1.90)	2.13 (1.83 to 2.48)

See eTable 2 and text for definitions of the study cohort, serum creatinine increases, and the adjusted model.

* Prescription coverage >90-days after retest

† Potassium > 6 mmol/L

Supplementary figure A. Cumulative mortality of incompletely monitored patients and according to detailed levels of creatinine increases following renin-angiotensin system blockade

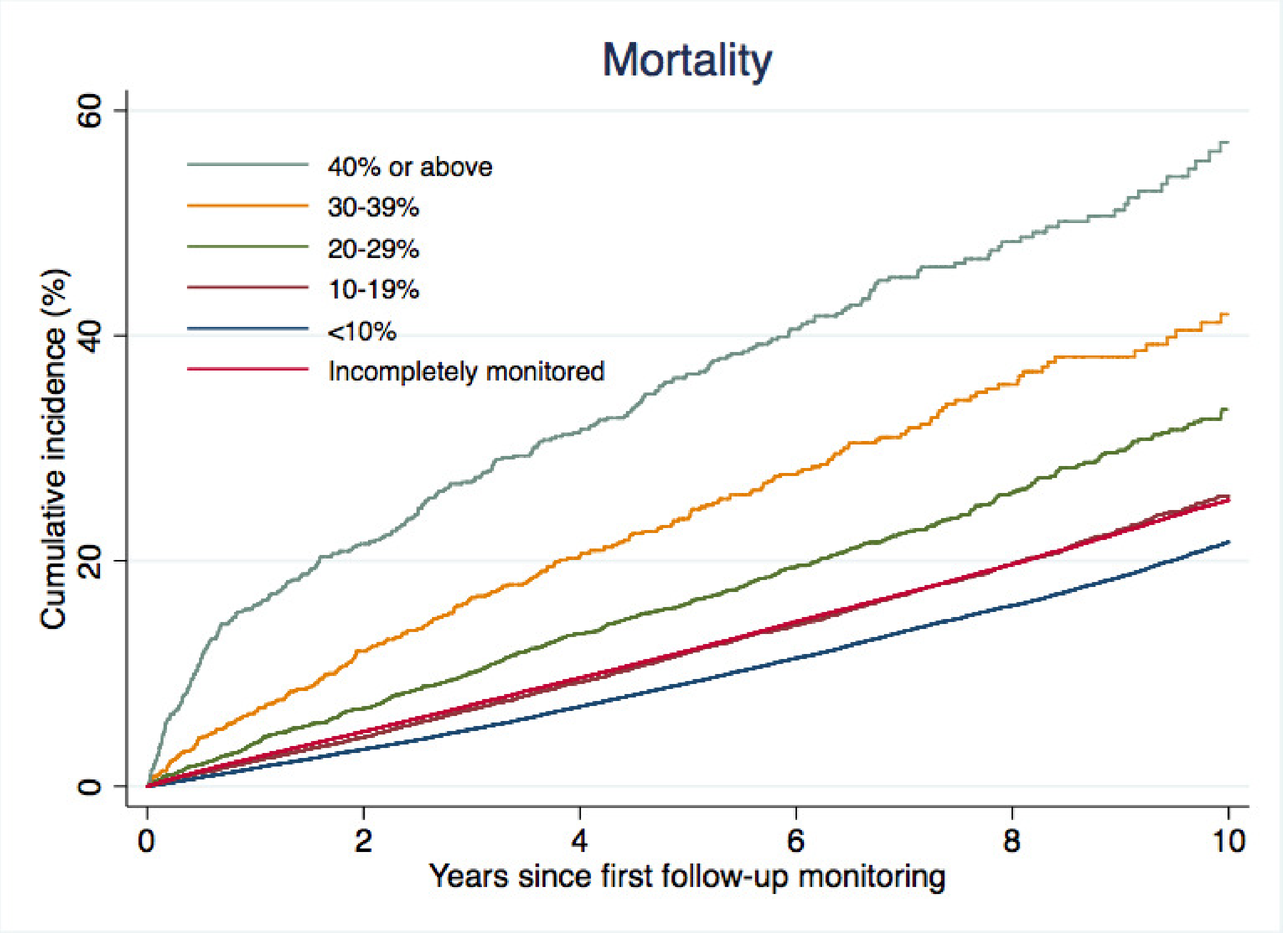

Supplement: Supplementary file 1 — Supplementary tables and figure [file schm035107.ww1.pdf]
